# Practical methods for ordinal data meta-analysis in stroke

**DOI:** 10.1186/1745-6215-14-S1-P120

**Published:** 2013-11-29

**Authors:** Ashma Krishan, James Weir, Gordon Murray, Brenda Thomas, Peter Sandercock, Steff Lewis

**Affiliations:** 1 University of Edinburgh, Edinburgh, UK

## 

Ordinal outcomes are common in medical research. However, in meta-analyses, they are routinely analysed as binary outcomes.

The aim of this study was to compare fixed effects meta-analyses using ordinal methods (proportional odds) with binary methods (binary logistic regression) and assess any changes in conclusions and gains in precision.

The Modified Rankin Scale (mRS) is a commonly used outcome measure in stroke trials. It comprises six disability levels, and death. We examined all 132 systematic reviews of interventions published by the Cochrane Stroke Group in the Cochrane Library, 2010 Issue 11, to find included trials that measured the mRS. The final analysis was based on 216 studies from 24 systematic reviews.

Studies reported mRS results to a varying level of detail, from binary only, to all seven possible categories. Initially, all studies were treated as if the outcome was binary. They were then analysed using three categories, four categories, and so on. If a study did not report enough detail for a given analysis, it was included using as much detail as possible.

The standard error (SE) of the estimated pooled common odds ratio for the three-category analysis was smaller than two categories (mean ratio of SEs=0.94). Four categories was marginally better than three (mean ratio of SEs=0.99) but adding further categories was not further beneficial. In 2/24 (8%) reviews, the conclusions changed from favouring control or treatment group to no evidence of a difference. In the remainder of reviews, the conclusions did not change.

Ordinal methods should be considered when performing meta-analyses.

